# The Moderating Role of Resilience in the Personality-Mental Health Relationship During the COVID-19 Pandemic

**DOI:** 10.3389/fpsyt.2021.745636

**Published:** 2021-10-21

**Authors:** Claire Pauly, Fabiana Ribeiro, Valerie E. Schröder, Laure Pauly, Rejko Krüger, Anja K. Leist, the CON-VINCE Consortium

**Affiliations:** ^1^Department of Translational Neuroscience, Luxembourg Centre for Systems Biomedicine, University of Luxembourg, Esch-Belval, Luxembourg; ^2^Department of Neurology, Parkinson Research Clinic, Centre Hospitalier de Luxembourg, Luxembourg, Luxembourg; ^3^Department of Social Sciences, Institute for Research on Socio-Economic Inequality, University of Luxembourg, Esch-sur-Alzette, Luxembourg; ^4^Transversal Translational Medicine, Luxembourg Institute of Health, Luxembourg, Luxembourg

**Keywords:** resilience, Big Five, personality, mental health, depression, COVID-19 pandemic

## Abstract

**Background:** Associations between personality traits and mental health outcomes (depression, anxiety, loneliness, and stress) have rarely been assessed in a population-representative sample of a high-income country during the COVID-19 pandemic. Additionally, as far as we know, the role of health and social behaviors as well as resilience in the personality-mental health relationship has yet to be explored.

**Methods:** A representative sample of 1,828 residents of Luxembourg filled in validated scales to assess personality traits and resilience, depressive symptoms, generalized anxiety, loneliness, and stress, indicating mental health, in mid-April 2020.

**Results:** Approximately 21% of the participants scored above the cut-off for moderate depression and moderate loneliness. Moderate anxiety and moderate stress were present in 6.2 and 0.3% of the participants, respectively. Higher-educated respondents and those living in higher-value housing reported better mental health. Agreeableness and conscientiousness were most consistently associated with better mental health; neuroticism was most consistently associated with worse mental health. Spending more time on social media was also associated with elevated levels of all four mental health outcomes. Social and health behaviors did not change the personality-mental health relationships. Resilience moderated some of the personality-mental health associations, most consistently in neuroticism.

**Conclusions:** Findings suggest educational and socioeconomic inequalities in mental health in a nationally representative sample during the COVID-19 confinement measures. Personality traits, particularly agreeableness, conscientiousness, and low neuroticism were associated with mental health. The moderating role of resilience in the personality-mental health relationship suggests intervention potential to improve mental health during periods of confinement.

## Introduction

With exponentially increasing numbers of COVID-19 infections across Europe, Luxembourg declared a crisis in mid-March 2020. Confinement measures were implemented, including mandatory home confinement, closure of schools, restaurants, and bars. Only necessary activities were maintained to limit social contacts to a maximum extent. These measures have been shown to increase psychological distress in the general population, and increase loneliness and isolation for older adults or vulnerable people as well as for family members not living in the same household (1, 2). In the general European population, approximately one quarter has experienced symptoms of anxiety. About one-third reported elevated symptoms of depression (32.4%) and stress (31.9%) (3, 4).

The role of personality in mental health has been investigated in several pre-pandemic studies, with the “Big Five” model being the most well-known model of personality (5). The five personality traits are neuroticism, extraversion, openness, agreeableness, and conscientiousness, known as the most basic dimensions of personality. Neuroticism refers to the vulnerability to emotional instability and self-consciousness. Openness refers to the extent to which new impressions and experiences are made. People with high scores on this dimension are creative and have broad interests (5). Extraversion refers to being active and interpersonal behavior. People with high scores on this dimension are sociable, active, focused on other people as well as receptive to ideas (5). Conscientiousness is the tendency toward dutifulness and competence.

High levels of neuroticism are linked to less emotional stability and more negative mental health outcomes such as depression, anxiety, distress, and irritability (6–8). High levels of neuroticism and extraversion are related to lower emotional stability leading to higher levels of stress due to higher levels of perceived threat and lower levels of efficacy to cope with the situation (9, 10). In contrast, conscientiousness is associated with better health and well-being.

There is first evidence on the associations between personality traits and mental health outcomes during the first year of the pandemic (10). Our study extends earlier research by testing to which extent health and social behaviors during the pandemic control measures were able to explain the personality-mental health relationship and explores the role of resilience as potential moderator in this relationship.

Aside from personality traits, a number of further sociodemographic and behavioral factors were associated with mental health during pandemic control measures. For instance, a systematic review carried out by Xiong et al. (11) showed that female sex, lower education, being single, unemployment, and increased exposure to social media were the most common risk factors for poorer mental health. Similarly, as shown before higher-educated participants were less likely to present depression and anxiety symptoms than respondents with no formal educational degree (12). In fact, education seems to be a protective factor as it influences income and professional status (13). It had been confirmed that the unemployed had higher stress and anxiety levels compared to those in full-time employment.

In addition, a relevant sociodemographic factor in the influence of COVID-19 confinement measures on mental health is the type of housing (14–16), in which respondents living in small apartments, characterized by scarce views and lack of outdoor spaces, had more severe depression symptoms, consequently influencing well-being and work productivity. In Luxembourg, living in a house (vs. apartment) is additionally a rather robust proxy for individual and parental wealth due to higher value of houses than apartments (17).

Even though older adults are considered a high-risk group for COVID-19, and that several studies indicated that social disconnection and distancing put older adults at a greater risk for mental health problems (18, 19), the pandemic seems to have a greater impact on younger adults (20, 21). Symptoms of anxiety and depression are more present in younger adults compared to older adults in the longitudinal follow-up (12). It is possible that, due to less developed coping skills, young adults have more difficulties in coping with the current situation than older adults (22). Furthermore, remote work or work loss could disrupt social connectedness and identity (22). Additionally, separation from friends and a feeling of detachment from schools and universities could also increase the levels of depression, anxiety, and stress (23, 24). These disruptions may put an already vulnerable group at greater risk for mental health outcomes, seeing those under the age of 25 are already experiencing higher levels of loneliness, which may be more devastating due to social distancing and isolation (25).

In this sense, the use of social media and technological devices, which has been ever increasing over the last two decades, could serve on the one hand as a bridge to maintain social contacts. On the other hand, however, an increase in the time spent in front of a screen could have a negative impact on well-being, and setting time limits of social media usage may be buffering this negative effect (26).

Finally, in a period of confinement, resilience should positively contribute to coping with this exceptional life situation. Indeed, resilience has been suggested as an important determinant for issues related to mental health during the COVID-19 pandemic in a number of studies (27, 28) as it mediates the relationship between personality traits and psychological functioning except for extraversion (28). Resilience is defined as the ability to return to a previous level of functioning after an adverse life event or experience (29). Earlier research has shown the possibility to improve resilience through interventions (30). However, to our knowledge, it has not been explored yet to which extent resilience may moderate the personality-mental health relationship.

We hypothesized that during the first wave of the COVID-19 pandemic, personality traits would be associated with mental health outcomes (levels of depressive symptoms, anxiety, stress, and loneliness), similarly to evidence from pre-pandemic studies. Further, we explored if differences in health and social behaviors would explain the relationships of the personality traits with the mental health outcomes. Finally, we also examined if resilience would moderate the personality-mental health relationships.

## Materials and Methods

### Study Design

The recruitment of the participants took place in the framework of the nationally-representative CON-VINCE study, aiming at evaluating the dynamics of the spread of the COVID-19 disease as well as getting insights into the mental health of the Luxembourgish population (31). The chosen sampling strategy for the representativeness of the Luxembourgish population was based on gender-, age-, and residency-stratification in collaboration with a specialized survey company (TNS-ILRES) (31).

The full study has been described in detail elsewhere (31). The online administered questionnaire consisted, among others, of socioeconomic and health indicators, and validated scales to assess depression, anxiety, loneliness, stress, and personality traits.

Given the multilingual characteristic of Luxembourg, we used questionnaires with translations to all the four languages (German, French, English, and Portuguese) (32–34) and applied in large multi-country surveys such as the Survey of Health, Aging and Retirement in Europe (35, 36). This was done to ensure that participants could choose their preferred language to answer the study questionnaires.

The study was approved by the national research ethics committee (Comité National d'Ethique de Recherche, CNER), under reference 202004/01, and by the Luxembourgish Ministry of Health under reference 831x6ce0d. Furthermore, the study has been submitted for registration on ClinicalTrials.gov (NCT04379297).

### Participants

A total of 1,828 participants were randomly selected based on three stratification variables (gender, electoral district, and age) in order to reach representativeness of the Luxembourgish population (18–79 years).

### Material

#### Mental Health Measures

- The Center for Epidemiologic Studies Depression Scale (CES-D) (37–39) assesses depressive symptoms within the general population. A score of ≥16 out of 60 is indicative of an increased risk for clinical depression (40). The CES-D had a Cronbach's alpha of 0.89, which shows a good internal consistency and reliability.
- The Generalized Anxiety Disorder Assessment (GAD) (41–44) measures the presence and severity of a generalized anxiety syndrome. The cut-off is 10 out of 21 for moderate anxiety. The GAD-7 showed good internal consistency and reliability (Cronbach's alpha of 0.88).
- The Three-item Loneliness Scale (45) is derived from the Revised UCLA Loneliness Scale (46) and measures perceived subjective isolation and feelings of loneliness. A score of ≥6 is indicative of loneliness. The UCLA showed poor internal consistency (Cronbach's alpha of 0.60).
- The Perceived Stress Scale-4 item version (PSS-4) (47) is a self-administered measurement of stress. A score of 6 or higher indicates high level of stress based on population norms (48). We detected an acceptable internal consistency and reliability for PSS (Cronbach's alpha of 0.68).
- The Brief Resilience Scale (BRS) (29, 49–51) assesses resilience. Scores ranging from 3 to 4.3 are considered as a normal level of resilience. A score <3 is indicative of low resilience, whereas a score >4.3 indicates high resilience. The BRS had a Cronbach's alpha of 0.82, which means good internal consistency and reliability.

#### Personality Characteristics

- The Big Five Inventory 10 (BFI-10) provides information regarding a person's personality (34, 52, 53). These personality dimensions (extraversion, agreeableness, conscientiousness, neuroticism, and openness) are each represented by two items; constituting an assessment tool of an overall total of 10 items. The BFI-10 items varied in terms of internal consistency and reliability. Cronbach's alpha was 0.66 for extraversion, 0.24 for agreeableness, 0.45 for conscientiousness, 0.63 for neuroticism, and 0.49 for openness.

#### Health and Social Behaviors

Behavioral aspects in the context of the COVID-19 pandemic were measured by quantifying the frequency of different activities and behaviors. Among these, we focused on the physical activity before and during the pandemic, screen time as well as social contact by means of technological devices. Respondents were asked to report if they were physically active before and during the pandemic as well as the hours per week of exercise at the two time points. Participants reported how many hours per day they spent surfing the internet, on social media (Facebook, Twitter, Instagram…), watching the news on TV, speaking with friends or family via phone or video calls, and on streaming platforms.

Demographic data, specifically age, gender, marital status, education, and type of housing (living in a house vs. living in an apartment) were entered as control variables.

### Statistical Analysis

The study data were managed by using the *ADA: the CON-VINCE Discovery Platform*. STATA version 16 (StataCorp LP, College Station, TX, USA) was used to conduct statistical analyses, with α levels set at 0.05. Firstly, we computed descriptive statistics for demographic and socioeconomic variables, as well as psychological outcomes stratified by age groups.

Secondly, bivariate correlations were calculated to assess the associations between the independent variables and the continuous mental health outcomes (depressive symptoms, loneliness, anxiety, and stress). Finally, we performed multivariate regression models. We report unstandardized beta regression coefficients that indicate the change in the dependent variable for a one-unit increase in the independent variable. In the first set of regressions, we explored the associations of personality traits with the four mental health outcomes in separate models, controlling for sociodemographic variables. To check that the associations of psychological scales results with personality traits remained significant after adjustment, we introduced one variable at a time in the regression model before estimating the complete model. In a second step, health and social behaviors were additionally included to test if they could explain the relationship between personality traits and mental health outcomes. In a separate set of analyses, resilience was added as a main term and as an interaction term with the personality traits, first one at a time. Since associations did not change when entering all personality traits and their interaction terms with resilience at once, only the model incorporating all interactions is presented. Another way to conceptualize resilience is to hypothesize it as a mediator in the personality-mental health relationship. Therefore, in a last step, a structural equation model was set up to test the mediating role of resilience, using maximum likelihood method, as recommend by previous literature (54), the model fitted the structural equation modeling criteria (root mean square error of approximation—RMSEA and standardized root mean square residual—SRMR <0.08, goodness of fit index—GFI and comparative fit index—CFI >0.90).

## Results

### Descriptive Results

After excluding 34 participants with missing values on sociodemographic and other variables of interest, data of a total of 1,828 participants were analyzed (51.1% women). Participants' mean age was 47.6 years (*SD* = 15.1), average number of schooling was 14.0 years (*SD* = 3.7). The majority of participants (65.1%) were married or living in a registered partnership. More than half of the participants lived in their own house (73.7%), except for people in their 30s (39%). Most of the participants were Luxembourgish nationals (85.2%, Table 1).

**Table 1:** Demographic characteristics, psychological measures, personality traits, and behavioral aspects by age groups.

	**18–29**	**30–39**	**40–49**	**50–59**	**60–69**	**70–84**	**All subjects**
Nr of subjects	258	359	395	365	277	174	1,828
Age (in years) (mean; std)	25.2 (2.8)	34.7 (2.9)	44.5 (2.9)	54.7 (2.8)	64.0 (2.7)	73.6 (3.4)	47.6 (15.1)
Gender; female (*n*; %)	202 (54.7%)	185 (51.5%)	215 (54.3%)	191 (52.2%)	151 (54.5%)	52 (30.0%)	935 (51.1%)
Married/Registered partnership (*n*; %)	48 (18.7%)	225 (62.7%)	300 (75.8%)	299 (81.7%)	190 (68.6%)	130 (74.7%)	1,192 (65.1%)
Years of education (mean; std)	15.02 (3.5)	14.6 (3.8)	14.2 (3.5)	13.3 (3.6)	13.0 (3.6)	13.4 (3.6)	14.0 (3.7)
Luxembourgish nationality (*n*; %)	240 (93.0%)	319 (88.9%)	316 (79.8%)	288 (78.7%)	233 (84.1%)	163 (93.7%)	1,559 (85.2%)
Living in a house (*n*; %)	160 (62.0%)	217 (39%)	318 (80.3%)	303 (82.8%)	214 (77.3%)	136 (78.2%)	1,348 (73.7%)
**Mental health measures**							
GAD-7	4.4 (4.3)	3.8 (3.6)	3.2 (3.2)	3.2 (3.9)	2.7 (3.0)	1.9 (2.7)	3.3 (3.6)
*≥10 (%)*	31 (12%)	28 (7.8%)	17 (4.3%)	24 (6.6%)	9 (3.2%)	5 (2.9%)	114 (6.2%)
CES-D	14.0 (9.3)	11.0 (8.4)	9.8 (7.8)	10.5 (9.5)	9.0 (7.2)	7.5 (5.7)	10.4 (8.4)
*≥16*	94 (36.4%)	82 (22.8%)	73 (18.4%)	76 (20.8%)	43 (15.5%)	16 (9.2%)	384 (21.0%)
UCLA	5.0 (1.3)	4.6 (1.4)	4.3 (1.3)	4.5 (1.4)	4.5 (1.4)	4.2 (1.1)	4.5 (1.4)
*≥6*	79 (30.6%)	78 (21.7%)	71 (17.9%)	79 (21.6%)	50 (18.1%)	26 (14.9%)	383 (20.9%)
PSS-4 items	5.8 (2.7)	4.8 (2.7)	4.6 (2.8)	4.6 (3.0)	4.4 (2.8)	4.0 (2.5)	4.7 (2.8)
<6	126 (48.8%)	217 (60.4%)	260 (65.7%)	236 (64.5%)	181 (65.3%)	121 (69.5%)	1,141 (62.3%)
*≥6*	132 (51.2%)	142 (39.6%)	135 (34.3%)	129 (35.5%)	96 (34.7%)	53 (30.5%)	687 (37.7%)
BRS	3.2 (0.5)	3.3 (0.5)	3.4 (0.5)	3.3 (0.6)	4.5 (3.4)	3.4 (0.5)	3.3 (0.5)
1.0–2.99 (low)	*70 (27.1%)*	73 (20.3%)	72 (18.2%)	69 (18.9%)	44 (15.9%)	18 (10.3%)	346 (18.9%)
3.0–4.3 (normal)	*183 (70.9%)*	276 (76.9%)	317 (80.1%)	289 (79.0%)	228 (82.3%)	152 (87.9%)	1,446 (79.0%)
4.31–5.0 (high)	*5 (1.9%)*	10 (2.8%)	7 (1.8%)	8 (2.2%)	5 (1.8%)	3 (1.7%)	38 (2.1%)
**Personality traits**							
Agreeableness	3.3 (0.7)	3.3 (0.7)	3.4 (0.7)	3.4 (0.7)	3.5 (0.7)	3.5 (0.7)	3.4 (0.7)
Conscientiousness	3.6 (0.8)	3.7 (0.7)	4.0 (0.7)	4.0 (0.7)	4.0 (0.8)	4.0 (0.7)	3.9 (0.8)
Extraversion	3.2 (1.0)	3.4 (1.0)	3.5 (0.9)	3.5 (0.9)	3.6 (0.9)	3.5 (0.9)	3.5 (0.9)
Neuroticism	2.8 (0.9)	2.7 (0.9)	2.7 (0.9)	2.6 (0.9)	2.5 (0.9)	2.3 (0.8)	2.6 (0.9)
Openness	3.4 (1.0)	3.4 (1.0)	3.4 (0.9)	3.5 (0.9)	3.5 (1.0)	3.5 (0.3)	3.4 (0.9)
**Behavioral aspects**							
Pre-pandemic physical activity (yes–no) (*n*; %)	190 (73.6%)	222 (61.8%)	239 (60.4%)	235 (64.2%)	177 (63.9%)	102 (58.6%)	1,165 (63.7%)
Hours/Week of physical activity before pandemic	6.3 (5.4)	4.6 (3.2)	4.8 (4.0)	5.5 (4.1)	7.1 (5.4)	6.6 (4.4)	5.7 (4.5)
Physical activity during pandemic (yes–no) (*n*; %)	186 (72.1%)	196 (54.6%)	205 (51.8%)	192 (52.5%)	137 (49.5%)	76 (43.7%)	992 (54.2%)
Hours/Week of physical activity during pandemic	6.1 (4.4)	5.0 (3.6)	5.2 (3.8)	5.8 (3.7)	7.4 (4.9)	7.3 (4.1)	5.9 (4.1)
Hours/Day spent on the Internet	3.5 (3.0)	2.7 (3.0)	2.6 (3.0)	2.5 (2.9)	2.2 (2.4)	2.8 (4.1)	2.7 (3.0)
Hours/Day spent on social media	3.4 (3.7)	1.8 (2.8)	1.7 (2.5)	1.4 (2.1)	1.3 (2.4)	0.8 (2.3)	1.8 (2.8)
Hours/Day spent on Television to watch news	1.3 (2.1)	1.6 (2.4)	1.6 (2.6)	1.8 (2.6)	2.0 (2.5)	2.4 (2.6)	1.6 (2.4)
Hours/Day spent on phone or video call	2.1 (2.6)	1.7 (2.2)	1.6 (2.2)	1.6 (2.3)	1.6 (2.3)	1.6 (2.5)	1.7 (2.2)
Hours/Day spent on streaming platforms	3.5 (3.8)	1.8 (3.0)	1.9 (3.1)	1.5 (2.7)	0.7 (1.2)	0.5 (2.1)	1.8 (3.0)

*GAD-7, generalized anxiety disorder-7; CES-D, Center for epidemiologic studies-depression; UCLA, UCLA three-item loneliness scale; PSS, perceived stress scale; BRS, brief resilience scale; STD, standard deviation; na, not available*.

Regarding psychological variables, 687 participants (37.7%) indicated a high level of stress, and 114 participants (6.2%) displayed moderate anxiety levels. Significant levels of moderate and higher depression as well as loneliness were found in 384 (21%) and 383 (20.9%) participants, respectively. Thousand four hundred and forty-six participants (79%) had typical levels of resilience, while 346 (18.9%) reported low resilience levels. In the age range 18–29, more people scored above the cut-off for moderate depression (36.4%), anxiety (12.0%), and loneliness (30.6%) than the other age groups. In the same age range, participants reported higher levels of stress (48.8%) than in the other age ranges. The prevalence of moderate levels of psychological distress was stable across the age ranges.

Frequency of physical activity significantly decreased during the confinement compared to before the pandemic (63.7 vs. 54.2%; *p* < 0.001) in all age groups. However, the time spent on physical activity significantly increased during the pandemic for all the age ranges (*p* < 0.001). Moreover, young adults (18–29 years) spent more hours per day on the internet (*M* = 3.5, *SD* = 3.0), on social media (*M* = 3.4, *SD* = 3.7), on the phone (*M* = 2.1, *SD* = 2.6), and on streaming platforms (*M* = 3.5; *SD* = 3.8) compared to the other age groups. In the other age ranges, mean hours spent on technical devices remained stable (Table 1). Additional analyses showed no associations between annual income brackets and mental health outcomes.

### Bivariate Correlations

As we expected, anxiety was positively correlated with depressive symptoms (*r* = 0.79, *p* < 0.001), stress (*r* = 0.16, *p* < 0.001) as well as with loneliness (*r* = 0.44, *p* < 0.001). Depressive symptoms were positively correlated with loneliness (*r* = 0.52, *p* < 0.001), and with stress (*r* = 0.76, *p* = 0.001).

We found positive correlations between agreeableness and resilience (*r* = 0.14, *p* < 0.001) as well as between conscientiousness and resilience and between extraversion and resilience (*r* = 0.78, *p* = 0.001, and *r* = 0.98, *p* < 0.001, respectively). A negative correlation has been established between resilience and neuroticism (*r* = −0.28, *p* < 0.001).

### Multivariate Regression Models

In the first set of analyses, depression, anxiety, loneliness, and stress served as dependent variables, and we controlled for age, gender, education, marital status, and type of housing.

Here, higher agreeableness was associated with lower depression (*B* = −0.58; *SE* = 0.25), lower anxiety (*B* = −0.28; *SE* = 0.10), lower loneliness (*B* = −0.15; *SE* = 0.05), and lower stress (*B* = −0.41; *SE* = 0.09; Table 2). Higher conscientiousness was also associated with lower depression (*B* = −0.97; *SE* = 0.24), lower loneliness (*B* = −0.13; *SE* = 0.04), and lower stress (*B* = −0.32; *SE* = 0.08). Low extraversion predicted higher stress (*B* = −0.22; *SE* = 0.67). Conversely, high neuroticism was associated with higher depression (*B* = 3.39; *SE* = 0.20), higher anxiety (*B* = 1.72; *SE* = 0.09), higher loneliness (*B* = 0.34; *SE* = 0.04), and higher stress (*B* = 1.05; *SE* = 0.71). Higher openness was associated with higher depression (*B* = 0.57; *SE* = 0.19), higher anxiety (*B* = 0.28; *SE* = 0.08), and higher loneliness (*B* = 0.08; *SE* = 0.03; Table 2). Respondents with more years of education had less anxiety (*B* = −0.05; *SE* = 0.02). Participants living in a house showed fewer depression symptoms than those living in an apartment (*B* = −1.58; *SE* = 0.40). Furthermore, marital status played a role: higher stress symptoms were found in respondents who reported being single (*B* = 0.43; *SE* = 0.19) or widowed (*B* = 1.01; *SE* = 0.40), while higher loneliness symptoms were associated with being single (*B* = 0.40; *SE* = 0.09), divorced (*B* = 0.39; *SE* = 0.11), or widowed (*B* = 0.45; *SE* = 0.20). The percentage of variance (*R*^2^) explained by the independent variables in the models for stress, depression, anxiety, and loneliness scales were 23, 27, 29, and 14%, respectively.

**Table 2:** Associations of mental health and personality traits outcomes, controlling for sociodemographic variables.

	**Perceived stress**			**Depression**			**Anxiety**			**Loneliness**		
	***B***	***SE***	***P***	***B***	***SE***	***P***	***B***	***SE***	***P***	***B***	***SE***	***P***
Agreeableness	**−0.41**	**0.085**	**0.000**	**−0.578**	**0.246**	**0.019**	**−0.271**	**0.104**	**0.009**	**−0.147**	**0.043**	**0.001**
Conscientiousness	**−0.32**	**0.082**	**0.000**	**−0.964**	**0.237**	**0.000**	−0.039	0.100	0.695	**−0.127**	**0.042**	**0.002**
Extraversion	**−0.22**	**0.67**	**0.001**	−0.353	0.193	0.067	−0.044	0.081	0.586	0.032	0.034	0.351
Neuroticism	**1.05**	**0.705**	**0.000**	**3.394**	**0.203**	**0.000**	**1.721**	**0.085**	**0.000**	**0.341**	**0.036**	**0.000**
Openness	0.05	0.652	0.445	**0.574**	**0.187**	**0.002**	**0.280**	**0.079**	**0.000**	**0.081**	**0.033**	**0.015**
**Age groups**												
18–29 (reference)												
30–39	**−0.64**	**0.086**	**0.004**	**−1.527**	**0.642**	**0.018**	−0.442	0.271	0.103	−0.133	0.113	0.239
40–49	**−0.6**	**0.082**	**0.011**	**−1.696**	**0.681**	**0.013**	**−0.867**	**0.287**	**0.003**	**−0.345**	**0.120**	**0.004**
50–59	**−0.52**	**0.067**	**0.034**	−0.693	0.707	0.327	**−0.794**	**0.298**	**0.008**	−0.132	0.124	0.290
60–69	**−0.65**	**0.07**	**0.012**	**−2.004**	**0.746**	**0.007**	**−1.201**	**0.315**	**0.000**	−0.114	0.131	0.385
70–4	**−0.82**	**0.065**	**0.005**	**−2.388**	**0.844**	**0.005**	**−1.415**	**0.356**	**0.000**	−0.249	0.148	0.094
**Sex**												
Female (reference)												
Male	**−0.3**	**0.12**	**0**	**−2.197**	**0.361**	**0.000**	**−0.786**	**0.152**	**0.000**	**−0.241**	**0.063**	**0.000**
School Years	−0.05	0.02	0.28	−0.041	0.047	0.385	**−0.051**	**0.020**	**0.012**	−0.010	0.008	0.235
**Marital status**												
Married/Registered partnership (reference)												
Single	**0.43**	**0.19**	**0.02**	**2.032**	**0.532**	**0.000**	0.093	0.224	0.678	**0.400**	**0.094**	**0.000**
Divorced	0.43	0.22	0.06	1.200	0.646	0.063	0.098	0.272	0.719	**0.391**	**0.113**	**0.001**
Widow	**1.01**	**0.40**	**0.01**	2.140	1.152	0.063	0.215	0.486	0.658	**0.454**	**0.202**	**0.025**
Other Status	0.49	0.38	0.20	0.723	1.085	0.506	0.446	0.458	0.330	0.370	0.191	0.052
**Housing**												
Apartment (reference)												
House	−0.10	0.14	0.48	**−1.584**	**0.405**	**0.000**	−0.279	0.171	0.103	0.001	0.071	0.989
Others	0.96	0.85	0.26	2.455	2.451	0.317	1.302	1.034	0.208	−0.154	0.431	0.721

*B, unstandardized regression coefficient; SE, standard error; p, P-value. Bold letters indicate significant coefficients with p smaller 0.05*.

By further including health and social behaviors in the second set of analyses, we found that the more time people spent on social media, the more they experienced depression (*B* = 0.19; *SE* = 0.08), anxiety (*B* = 0.08; *SE* = 0.04), loneliness (*B* = 0.03; *SE* = 0.02), and stress (*B* = 0.07; *SE* = 0.03; Table 3). Other social and health behaviors were not significantly associated with the mental health outcomes and did not modify the personality-mental health relationships. The independent variables explained a total of 24% percentage of variance in the model for stress, 28% in the model for depression, 30% for anxiety, and 14% for loneliness.

**Table 3:** Associations of personality traits, mental health outcomes and social and behavioral aspects, controlling for sociodemographic variables.

	**Perceived Stress**			**Depression**			**Anxiety**			**Loneliness**		
	***B***	***SE***	***P***	***B***	***SE***	***P***	***B***	***SE***	***P***	***B***	***SE***	***P***
Agreeableness	**−0.394**	**0.087**	**0.000**	**−0.602**	**0.250**	**0.016**	**−0.256**	**0.105**	**0.015**	**−0.144**	**0.045**	**0.001**
Conscientiousness	**−0.284**	**0.085**	**0.001**	**−0.782**	**0.243**	**0.001**	0.010	0.102	0.923	**−0.117**	**0.043**	**0.007**
Extraversion	**−0.247**	**0.069**	**0.000**	**−0.441**	**0.197**	**0.025**	−0.071	0.083	0.389	0.004	0.035	0.907
Neuroticism	**1.048**	**0.072**	**0.000**	**3.391**	**0.206**	**0.000**	**1.733**	**0.087**	**0.000**	**0.332**	**0.037**	**0.000**
Openness	0.013	0.067	0.848	**0.450**	**0.191**	**0.018**	**0.237**	**0.080**	**0.003**	**0.083**	**0.034**	**0.015**
**Age groups**												
18–29 (reference)												
30–39	**−0.531**	**0.229**	**0.020**	−1.216	0.653	0.063	−0.317	0.275	0.249	−0.073	0.117	0.533
40–49	**−0.542**	**0.245**	**0.027**	**−1.409**	**0.699**	**0.044**	**−0.748**	**0.294**	**0.011**	**−0.259**	**0.125**	**0.038**
50–59	−0.391	0.256	0.126	−0.263	0.731	0.719	**−0.662**	**0.307**	**0.031**	−0.066	0.130	0.613
60–69	−0.530	0.274	0.053	**−1.609**	**0.782**	**0.040**	**−1.064**	**0.329**	**0.001**	−0.032	0.140	0.818
70–84	**−0.695**	**0.313**	**0.026**	**−1.978**	**0.894**	**0.027**	**−1.351**	**0.376**	**0.000**	−0.169	0.160	0.290
**Sex**												
Female (reference)												
Male	**−0.272**	**0.130**	**0.037**	**−2.232**	**0.371**	**0.000**	**−0.710**	**0.156**	**0.000**	**−0.217**	**0.066**	**0.001**
School Years	**−0.050**	**0.017**	**0.003**	−0.028	0.048	0.558	−0.035	0.020	0.084	−0.008	0.009	0.363
**Marital status**												
Married/ registered partnership (reference)												
Single	**0.411**	**0.188**	**0.029**	**2.056**	**0.538**	**0.000**	0.095	0.226	0.675	**0.399**	**0.096**	**0.000**
Divorced	0.438	0.228	0.054	1.066	0.651	0.102	0.045	0.274	0.871	**0.347**	**0.116**	**0.003**
Widow	**1.074**	**0.415**	**0.010**	**2.488**	**1.186**	**0.036**	0.164	0.499	0.743	**0.511**	**0.212**	**0.016**
Other Status	0.440	0.386	0.254	0.766	1.102	0.487	0.435	0.464	0.349	0.378	0.197	0.055
**Housing**												
Apartment (reference)												
House	−0.105	0.143	0.466	**−1.468**	**0.410**	**0.000**	−0.282	0.172	0.102	−0.008	0.073	0.909
Others	1.022	0.851	0.230	2.626	2.431	0.280	1.332	1.023	0.193	−0.111	0.434	0.797
**Social and behavioral aspects**												
Pre-COVID-19 exercise (ref. no exercise)	−0.159	0.159	0.318	0.263	0.454	0.563	−0.166	0.191	0.386	−0.002	0.081	0.978
Current exercise (ref. no exercise)	0.077	0.154	0.617	−0.507	0.439	0.248	−0.137	0.185	0.459	0.057	0.078	0.464
Hours spent daily												
On the internet	−0.016	0.026	0.536	0.018	0.073	0.802	−0.025	0.031	0.416	−0.004	0.013	0.774
On social media	**0.068**	**0.029**	**0.020**	**0.190**	**0.083**	**0.023**	**0.080**	**0.035**	**0.023**	**0.030**	**0.015**	**0.045**
News and TV	0.023	0.031	0.454	−0.004	0.089	0.961	0.038	0.038	0.306	0.001	0.016	0.938
Phone calls	−0.020	0.033	0.538	0.048	0.094	0.608	0.034	0.039	0.388	0.012	0.017	0.491
Streaming	0.011	0.024	0.660	0.000	0.069	0.995	−0.009	0.029	0.770	−0.012	0.012	0.323

*B, unstandardized regression coefficient; SE, standard error, p, P-value. Bold letters indicate significant coefficients with p smaller 0.05*.

In the next step, the models additionally included resilience, which was strongly and negatively associated with all four mental health outcomes (Table 4). Including resilience, the direction and significance of associations of stress, anxiety, and loneliness with personality traits did not change. Depression, agreeableness, and extraversion were no longer significantly associated with resilience, suggesting that resilience was more strongly associated with mental health than the personality traits. The independent variables explained 24% in the model for stress, 30% in the model for depression, 31% in the model for anxiety, and 15% for loneliness.

**Table 4:** Associations of mental health outcomes, personality traits, and resilience, controlling for sociodemographic variables.

	**Perceived stress**			**Depression**			**Anxiety**			**Loneliness**		
	***B***	***SE***	***P***	***B***	***SE***	***P***	***B***	***SE***	***P***	***B***	***SE***	***P***
Agreeableness	**−0.381**	**0.085**	**0.000**	−0.451	0.243	0.064	**−0.222**	**0.103**	**0.031**	**−0.134**	**0.043**	**0.002**
Conscientiousness	**−0.313**	**0.082**	**0.000**	**−0.934**	**0.233**	**0.000**	−0.028	0.099	0.780	**−0.124**	**0.041**	**0.003**
Extraversion	**−0.203**	**0.067**	**0.002**	−0.271	0.190	0.154	−0.013	0.080	0.874	0.040	0.034	0.232
neuroticism	**0.959**	**0.072**	**0.000**	**3.052**	**0.204**	**0.000**	**1.590**	**0.086**	**0.000**	**0.305**	**0.036**	**0.000**
Openness	0.026	0.065	0.688	**0.484**	**0.185**	**0.009**	**0.246**	**0.078**	**0.002**	**0.071**	**0.033**	**0.031**
**Age groups**												
18–29 (reference)												
30–39	**−0.597**	**0.222**	**0.007**	**−1.373**	**0.632**	**0.030**	−0.383	0.267	0.152	−0.117	0.112	0.299
40–49	**−0.560**	**0.235**	**0.017**	**−1.529**	**0.670**	**0.023**	**−0.803**	**0.283**	**0.005**	**−0.327**	**0.119**	**0.006**
50–59	**−0.496**	**0.244**	**0.042**	−0.595	0.696	0.393	**−0.757**	**0.294**	**0.010**	−0.121	0.124	0.327
60–69	**−0.599**	**0.257**	**0.020**	**−1.814**	**0.735**	**0.014**	**−1.128**	**0.311**	**0.000**	−0.094	0.130	0.473
70–84	**−0.771**	**0.291**	**0.008**	**−2.193**	**0.831**	**0.008**	**−1.340**	**0.351**	**0.000**	−0.228	0.148	0.122
**Sex**												
Female (reference)												
Male	**−0.275**	**0.125**	**0.027**	**−2.106**	**0.355**	**0.000**	**−0.752**	**0.150**	**0.000**	**−0.231**	**0.063**	**0.000**
School Years	**−0.050**	**0.016**	**0.002**	−0.033	0.047	0.486	**−0.047**	**0.020**	**0.017**	−0.009	0.008	0.279
**Marital status**												
Married/ registered partnership (reference)												
Single	**0.406**	**0.184**	**0.027**	**1.945**	**0.524**	**0.000**	0.060	0.222	0.786	**0.391**	**0.093**	**0.000**
Divorced	0.438	0.223	0.049	**1.239**	**0.635**	**0.051**	0.113	0.269	0.674	**0.395**	**0.113**	**0.000**
Widow	**1.050**	**0.397**	**0.008**	**2.304**	**1.134**	**0.042**	0.278	0.479	0.562	**0.472**	**0.201**	**0.019**
Other Status	0.555	0.374	0.138	0.979	1.069	0.360	0.544	0.452	0.228	**0.398**	**0.190**	**0.036**
**Housing**												
Apartment (reference)												
House	−0.092	0.140	0.510	−1.559	0.398	0.000	−0.269	0.169	0.110	0.004	0.071	0.959
Others	0.758	0.846	0.370	1.692	2.414	0.484	1.009	1.021	0.323	−0.235	0.429	0.584
Resilience	**−0.672**	**0.115**	**0.000**	**−2.545**	**0.328**	**0.000**	**−0.976**	**0.139**	**0.000**	**−0.270**	**0.058**	**0.000**

*B, unstandardized regression coefficient; SE, standard error; p, P-value. Bold letters indicate significant coefficients with p smaller 0.05*.

Next, testing the moderating role of resilience on mental health outcomes, resilience was interacted with the personality traits first one at a time, then in a joint model. As the associations were similar, only the full model containing all five interactions is reported (Table 5). Here, in more conscientious respondents, higher resilience was associated with higher levels of stress (*B* = 0.316, *SE* = 0.148, *p* = 0.033). In more neurotic respondents, higher resilience was associated with lower levels of stress (*B* = −0.259, *SE* = 0.124, *p* = 0.037). Higher resilience was also associated with lower levels of depression and anxiety, respectively, in more neurotic respondents (depression: *B* = −1.798, *SE* = 0.349, *p* = 0.000; anxiety: *B* = −0.989, *SE* = 0.147, *p* = 0.000). Finally, in more open respondents, higher resilience was associated with lower levels of depression and anxiety (depression: *B* = −1.175, *SE* = 0.344, *p* = 0.001; anxiety: *B* = −0.431, *SE* = 0.145, *p* = 0.003).

**Table 5:** Associations of personality traits, resilience and mental health outcomes, controlling for sociodemographic variables.

	**Perceived stress**			**Depression**			**Anxiety**			**Loneliness**		
	***B***	***SE***	***P***	***B***	***SE***	***P***	***B***	***SE***	***P***	***B***	***SE***	***P***
Agreeableness	−0.048	0.529	0.928	−2.450	1.492	0.101	−0.723	0.629	0.251	−0.330	0.269	0.220
Conscientiousness	**−1.352**	**0.498**	**0.007**	**−3.471**	**1.404**	**0.014**	−0.854	0.592	0.150	0.047	0.253	0.853
Extraversion	−0.011	0.408	0.978	−1.556	1.150	0.176	0.037	0.485	0.940	−0.160	0.207	0.439
Neuroticism	**1.782**	**0.405**	**0.000**	**8.776**	**1.141**	**0.000**	**4.749**	**0.481**	**0.000**	**0.689**	**0.205**	**0.001**
Openness	0.482	0.411	0.241	**4.435**	**1.160**	**0.000**	**1.695**	**0.489**	**0.001**	0.250	0.209	0.231
Resilience	−0.152	1.044	0.884	0.157	2.942	0.957	1.828	1.241	0.536	0.036	0.529	0.946
Agreeableness × Resilience	−0.102	0.159	0.520	0.601	0.449	0.181	0.148	0.189	0.435	0.059	0.081	0.465
Conscientiousness × Resilience	**0.316**	**0.148**	**0.033**	0.785	0.417	0.060	0.261	0.176	0.138	−0.050	0.075	0.507
Extraversion × Resilience	−0.059	0.122	0.629	0.384	0.343	0.264	−0.017	0.145	0.908	0.061	0.062	0.327
Neuroticism × Resilience	**−0.2 59**	**0.124**	**0.0 37**	**−1.798**	**0.349**	**0.000**	**−0.989**	**0.147**	**0.000**	−0.120	0.063	0.057
Openness × Resilience	−0.135	0.122	0.267	**−1.175**	**0.344**	**0.001**	**−0.431**	**0.145**	**0.003**	−0.053	0.062	0.390

*Values in bold represent statistically significant associations. B, unstandardized regression coefficient; SE, standard error; p, P-value. The model includes the covariates age group, sex, school years, marital status, and type of housing*.

### Structural Equation Modeling

**Figure 1:**
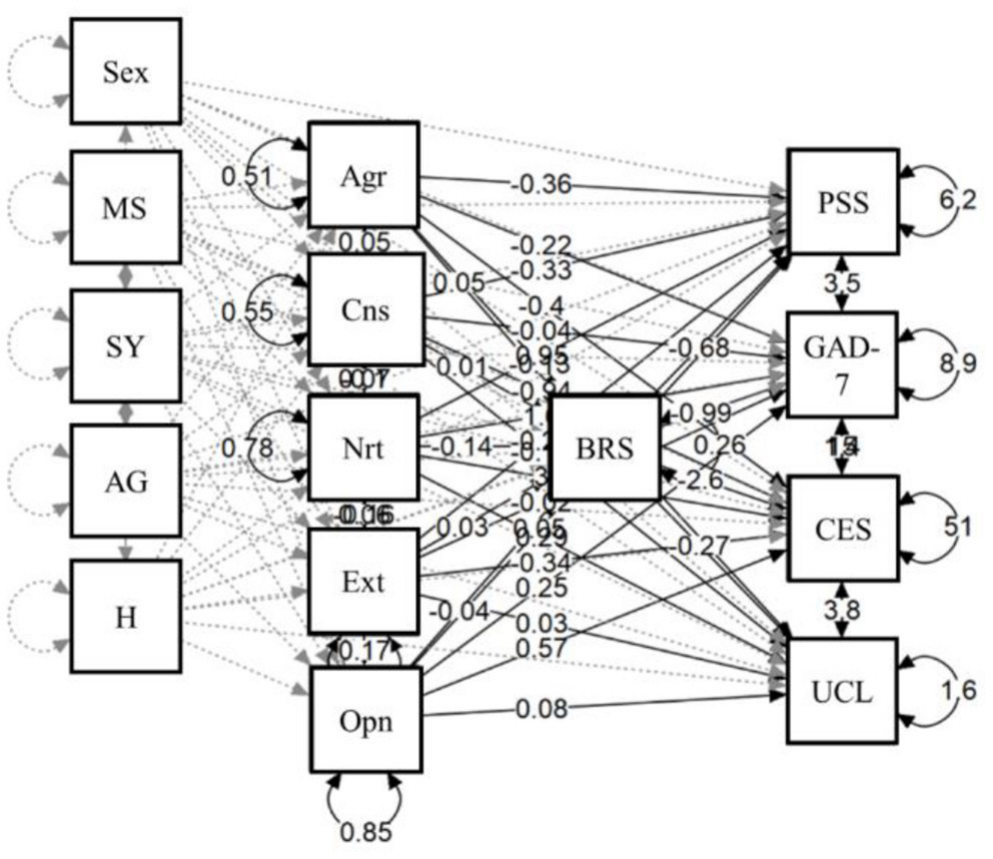
Resilience as a mediator in the personality-mental health relationship. MS, marital status; SY, school years; AG, age groups; H, housing; Agr, agreeableness; Cns, conscientiousness; Nrt, neuroticism; Ext, extraversion; Opn, openness; BRS, brief resilience scale; GAD-7, generalized anxiety disorder-7; CES-D, center for epidemiologic studies-depression; UCLA, UCLA three-item loneliness scale; PSS, perceived stress scale.

In a last step, a structural equation model mediation analysis was carried out to test whether the resilience could explain the associations between personality traits and mental health during the pandemic, also controlling for sociodemographic variables. The results showed that resilience plays a role as a mediator in mental health outcomes. More specifically, higher levels of resilience in more extroverted people were associated with higher levels of perceived stress (*B* = 0.355; SE = 0.058; *p* < 0.01), whereas they are associated with lower levels of anxiety (*B* = −0.089; SE = 0.037, *p* < 0.05), depression (*B* = −0.097; *SE* = 0.036, *p* < 0.01), and loneliness (*B* = −0.013; *SE* = 0.005, *p* < 0.05). Similarly, more resilient, and conscientious people showed higher levels of perceived stress (*B* = 0.025; *SE* = 0.010, *p* < 0.05), loneliness (*B* = 0.134; SE = 0.023, *p* < 0.01), and they tend to have lower levels of anxiety (*B* = −0.048; SE = 0.018, *p* < 0.01). Table 6 shows the indirect effects of the model, and Figure 1 shows the model tested.

**Table 6:** Structural equation modeling with resilience as mediator.

**Indirect effects**					**Estimate**	**Std. Error**	***z*-value**	***p***	**95% Confidence Interval**	
									**Lower**	**Upper**
**Agreeabl**	**→**	**BRS**	**→**	**PSS**	**−0.033**	**0.013**	**−2.547**	**0.011**	**−0.058**	**−0.008**
**Conscien**	**→**	**BRS**	**→**	**PSS**	**0.025**	**0.010**	**2.555**	**0.011**	**0.006**	**0.045**
**Neurotic**	**→**	**BRS**	**→**	**PSS**	**−0.034**	**0.014**	**−2.383**	**0.017**	**−0.061**	**−0.006**
**Extraver**	**→**	**BRS**	**→**	**PSS**	**0.355**	**0.058**	**6.129**	** <0.001**	**0.242**	**0.469**
Openness	→	BRS	→	PSS	−0.003	0.005	−0.726	0.468	−0.012	0.006
Agreeabl	→	BRS	→	GAD−7	−0.008	0.011	−0.730	0.466	−0.030	0.014
**Conscien**	**→**	**BRS**	**→**	**GAD-7**	**−0.048**	**0.018**	**−2.624**	**0.009**	**−0.084**	**−0.012**
**Neurotic**	**→**	**BRS**	**→**	**GAD-7**	**0.037**	**0.014**	**2.633**	**0.008**	**0.009**	**0.064**
**Extraver**	**→**	**BRS**	**→**	**GAD-7**	**−0.089**	**0.037**	**−2.409**	**0.016**	**−0.162**	**−0.017**
**Openness**	**→**	**BRS**	**→**	**GAD-7**	**0.037**	**0.009**	**4.150**	** <0.001**	**0.019**	**0.054**
**Agreeabl**	**→**	**BRS**	**→**	**CES**	**0.092**	**0.018**	**5.037**	** <0.001**	**0.056**	**0.128**
Conscien	→	BRS	→	CES	−0.012	0.016	−0.731	0.465	−0.044	0.020
**Neurotic**	**→**	**BRS**	**→**	**CES**	**−0.127**	**0.048**	**−2.660**	**0.008**	**−0.221**	**−0.034**
**Extraver**	**→**	**BRS**	**→**	**CES**	**0.097**	**0.036**	**2.669**	**0.008**	**0.026**	**0.169**
**Openness**	**→**	**BRS**	**→**	**CES**	**−0.009**	**0.004**	**−2.216**	**0.027**	**−0.017**	**−0.001**
**Agreeabl**	**→**	**BRS**	**→**	**UCLA**	**−0.023**	**0.010**	**−2.325**	**0.020**	**−0.043**	**−0.004**
**Conscien**	**→**	**BRS**	**→**	**UCLA**	**0.134**	**0.023**	**5.727**	** <0.001**	**0.088**	**0.179**
Neurotic	→	BRS	→	UCLA	−0.032	0.043	−0.732	0.464	−0.117	0.053
**Extraver**	**→**	**BRS**	**→**	**UCLA**	**−0.013**	**0.005**	**−2.405**	**0.016**	**−0.024**	**−0.002**
**Openness**	**→**	**BRS**	**→**	**UCLA**	**0.010**	**0.004**	**2.412**	**0.016**	**0.002**	**0.018**

*Delta method standard errors, normal theory confidence intervals, ML estimator. Bold letters indicate significant coefficients with p smaller 0.05*.

## Discussion

### Explanation of Findings

The aim of this study was to investigate the associations between personality traits and mental health in a period of confinement during the first wave of the COVID-19 pandemic in a nationally representative sample, and to which extent social and health behaviors would explain the personality-mental health relationships. We further explored the moderating role of resilience in the personality-mental health relationship.

The CON-VINCE participants reported higher levels of moderate depression compared to another study in Luxembourg carried out before the pandemic (55) and comparatively high levels of loneliness. The prevalence of depressive symptoms during the pandemic was in line with previous findings (10). Comparing the findings to another European country, Luxembourg, at that time point, only had few confirmed COVID-19 cases and a low prevalence of the SARS-CoV-2 virus (31), and had comparatively lower anxiety levels than Austria at the same time (56).

Confirming earlier findings (11), higher levels of education were associated with fewer depression symptoms. Higher education seems to be related to greater stability and access to employment opportunities, which is associated with higher well-being, on the other hand, people with lower education are the ones that might experience the first impact of the economic crisis due to restriction measures (57). As reported in other countries, such as Italy (14) and France (58) we also found that the type of housing was associated with mental health, with respondents living in an apartment reporting increased depression symptoms. This may be due to feeling more confined when living in a (usually less spacious) apartment. However, as living in a house is a robust proxy for wealth in this setting, also more robust than annual income for which we did not find associations with mental health, these findings may also point to socioeconomic inequalities in mental health.

Younger respondents presented higher scores on depression and anxiety scales as well as higher feelings of loneliness and perceived stress than other age groups in the CON-VINCE study (12) in line with earlier studies (3, 16). Testing the association of personality with mental health, personality traits were associated with mental health outcomes independent of age and other socio-demographic confounders.

The positive correlations between resilience and personality traits showed that the higher the participants scored on the traits agreeableness, conscientiousness, and extroversion, the higher their resilience was. The negative correlation between resilience and neuroticism showed that more neurotic participants were less resilient.

### Associations of Personality Traits With Mental Health Measures

We found associations between neuroticism and impaired mental health such as depression, anxiety, loneliness, and stress, in line with findings from pre-pandemic studies (5, 59). Neuroticism has been found to be linked to negative affect in a German sample in the first wave of the pandemic (60). Resilience was negatively linked to neuroticism, confirming previous results of neuroticism being a risk factor for anxiety and depressive symptoms (61, 62).

While conscientious people would search for distractions or act to resolve their problems, respondents with higher scores of neuroticism may ruminate as a way to react to stressful events, which may increase vigilance for possible threats in turn associated with stress and anxiety symptoms (63). In addition, if anxiety symptoms are not treated, it can potentially increase depression levels by the intense ruminating on negative expectations and persistent worries (64). In contrast to the literature, we found that people with higher levels of openness experienced higher levels of depression, anxiety, and loneliness. However, this result could be explained by different facets of this personality trait, especially fantasy, which may lead to experiencing higher discrepancies between idealized and actual self- or world-states and come with a higher risk for depression (65). We found that people scoring high in conscientiousness are experiencing lower stress levels inconsistent with pre-pandemic studies (66, 67), suggesting that higher conscientiousness may be beneficial in adjusting to pandemic control measures and accepting the new situation.

We found respondents with higher levels of extraversion to be experiencing lower levels of stress, in line with previous findings (68). Participants scoring lower on agreeableness were experiencing higher stress levels, again confirming previous findings (69). Interestingly, we could confirm a protective role of agreeableness and extraversion on perceived stress, personality traits which may have helped to activate beneficial coping strategies as suggested earlier (11). However, in this study, extraversion was not associated with lower anxiety (10), pointing to a more important role of agreeableness.

### Health and Social Behaviors in the Personality-Mental Health Relationship

The social and health behaviors investigated in this study, specifically the daily time spent on the internet, news, and TV, phone calls as well as on streaming platforms and by the physical activity before and during the pandemic unexpectedly had limited value to explain levels of stress, depression, anxiety, and loneliness, except time spent on social media. Further, the investigated social and health behaviors did not substantially change the personality-mental health relationships. One possible explanation is that the items to assess time spent with different behaviors may not have been able to capture fine-grained differences in the behaviors, or that behaviors resulted from different motivations or took place in different contexts (e.g., streaming alone vs. as a family or couple activity).

We found more time spent on social media to be related with stress, depression, anxiety, and loneliness, but not with personality traits. Our results provide insight into the negative role of social media in mental health, and confirm earlier findings (26) in the pandemic context. Due to the cross-sectional nature of our study, there is, however, the possibility of reverse causality. We believe that different problematic contents in social media, such as fake news and conspiracy theories, or overly positive self-portrayals of others, could lead to increased levels of stress and anxiety. We also believe that social media may provide reminders of social contacts, foreign places, and activities that were missed or not possible to reach during the confinement. This could have led to increased depressive symptoms and feelings of loneliness.

### Moderating Role of Resilience

We found that higher resilience was associated with lower levels of depression, stress, anxiety, and loneliness, in line with previous research (70). Additional analyses showed that resilience moderated some of the personality-mental health associations, most consistently by lowering the negative associations of neuroticism with stress, depression, and anxiety in persons with higher resilience. Additionally, testing a potentially mediating role of resilience in the personality-mental health relationship with structural equation modeling confirmed the findings from the multivariate regression analyses. Due to the cross-sectional nature of the study, the potentially causal mediating role of resilience cannot be established and stricter research designs are needed to arrive at robust conclusions about the moderating vs. mediating role of resilience. However, the results point to an important contribution of resilience in mental health during the first wave of the COVID-19 pandemic. Considering that resilience has been shown to be modifiable through interventions (30), people with higher neuroticism may benefit from resilience interventions to improve coping strategies and reduce symptoms. Further, higher conscientiousness was associated with higher levels of stress in persons with higher resilience. A possible explanation could be that high levels of conscientiousness and high levels of resilience could be associated with a strong sense of having control over one's circumstances, which could go along with feelings of having to prepare or execute precautions not to lose control, which could, in turn, be associated with higher levels of stress.

### Strengths and Limitations

Strength of the study was a population-representative sample with a rapid data collection during the height of pandemic control measures in April 2020. As a limitation, we could not account objective self-isolation in the personality-mental health relationship as some participants were still commuting to work whereas others were staying at home, with possibly limited social exchange. Other factors such as asymptomatic COVID-19 infection or COVID-19 disease of the respondents or their loved ones were not included in the analyses; however, at this time prevalence of the SARS-CoV-2 virus was very low in Luxembourg (31).

The multilingual sample necessitated the use of a total of four languages. Psychometric testing of the scales was not possible in language-stratified subsamples as the sample sizes by language were too small. Not all scales have been validated in all four languages in previous studies, however, checks with several multilingual colleagues and back translation were undertaken to ensure proper translation. The multilingual assessment may have resulted in diminished internal consistencies of the scales.

Additionally, we did not have information on mental health status of the CON-VINCE participants before the pandemic. The prevalence of depressive disorders has risen sharply in the last years in Luxembourg, already before the pandemic. In 2017, 5.0% of the Luxembourgish population showed elevated levels of depressive symptoms (71), whereas in 2019, this number had already doubled (72). Elevated levels of negative psychological outcomes may thus be due to ongoing developments in the country, for instance, rising housing prices and increasing inequality (17). The CON-VINCE study sample is representative in terms of gender and age of Luxembourg residents, however migrants, which constitute a large share of the residents, may not be well-represented (73).

### Implications for Mental Health Interventions

Findings on higher depression levels during the pandemic control measures underline the need for psychological support during times of a pandemic. Additionally, in psychological interventions or psychotherapy, knowledge about the personality structure of trainees or clients could potentially benefit efforts to increase mental health during pandemic control measures.

Resilience, as shown in recent literature, is not only shaped by the individual, but originates from other factors such as family, community, and culture (74). Wang and Morav (75) showed that civic participation or living in a neighborhood with strong community support and friendship may also help build strong resilience. In this context, a recent study showed that the feeling of neighborhood attachment differs within ethnic minorities and that this feeling is more pronounced for the second-generation minorities (76). As Luxembourg is composed of almost 50% foreigners (77), public policies should also pay more attention to promote interethnic dialogues and residential integration in order to strengthen resilience throughout the community.

## Conclusion

The current study extends our knowledge about the role of resilience in the personality-mental health relationship and other protective and risk factors for mental health during the pandemic. By putting earlier knowledge on the possibilities to increase resilience through interventions to good use, this study's findings on the links between personality, resilience, and mental health could improve personalized interventions for psychological support in times of confinement.

## The CON-VINCE Consortium

Bruno Santos, Luxembourg Institute of Health, Strassen, Luxembourg; Bianca Dragomir, Luxembourg Institute of Health, Strassen, Luxembourg; Joëlle V. Fritz, Luxembourg Institute of Health, Strassen, Luxembourg; Claire Dording, Luxembourg Institute of Health, Strassen, Luxembourg; Marc P. O'Sullivan, Luxembourg Institute of Health, Strassen, Luxembourg; Anne-Marie Hanff, Luxembourg Institute of Health, Strassen, Luxembourg; Maxime Hansen, Department of Neurology, Parkinson Research Clinic, Centre Hospitalier de Luxembourg, Luxembourg, Luxembourg; Luxembourg Institute of Health, Strassen, Luxembourg; Markus Ollert, Luxembourg Institute of Health, Strassen, Luxembourg; Morgane Lemaire, Luxembourg Institute of Health, Strassen, Luxembourg; Nadia Beaupain, Luxembourg Institute of Health, Strassen, Luxembourg; Dirk Brenner, Luxembourg Institute of Health, Strassen, Luxembourg; Eleftheria Charalambous, Luxembourg Institute of Health, Strassen, Luxembourg; Emilie Charpentier, Luxembourg Institute of Health, Strassen, Luxembourg; Manuel Counson, Luxembourg Institute of Health, Strassen, Luxembourg; Olivia Domingues, Luxembourg Institute of Health, Strassen, Luxembourg; Lisa Hefele, Luxembourg Institute of Health, Strassen, Luxembourg; Gilles Iserentant, Luxembourg Institute of Health, Strassen, Luxembourg; Stéphanie Kler, Luxembourg Institute of Health, Strassen, Luxembourg; Mareike Neumann, Luxembourg Institute of Health, Strassen, Luxembourg; Jean-Yves Servais, Luxembourg Institute of Health, Strassen, Luxembourg; Chantal Snoeck, Luxembourg Institute of Health, Strassen, Luxembourg; Jonathan Turner, Luxembourg Institute of Health, Strassen, Luxembourg; Linda Hansen, Centre Hospitalier de Luxembourg, Luxembourg, Luxembourg; João M. Loureiro, Centre Hospitalier de Luxembourg, Luxembourg, Luxembourg; Béatrice Nicolaï, Centre Hospitalier de Luxembourg, Luxembourg, Luxembourg; Alexandra Schweicher, Centre Hospitalier de Luxembourg, Luxembourg, Luxembourg; Femke Wauters, Centre Hospitalier de Luxembourg, Luxembourg, Luxembourg; Lukas Pavelka, Luxembourg Centre for Systems Biomedicine, University of Luxembourg, Esch-Belval, Luxembourg; Department of Neurology, Parkinson Research Clinic, Centre Hospitalier de Luxembourg, Luxembourg, Luxembourg; Guilherme R. Meyers, Luxembourg Centre for Systems Biomedicine, University of Luxembourg, Esch-Belval, Luxembourg; Department of Neurology, Parkinson Research Clinic, Centre Hospitalier de Luxembourg, Luxembourg, Luxembourg; Amna Skrozic, Luxembourg Centre for Systems Biomedicine, University of Luxembourg, Esch-Belval, Luxembourg; Department of Neurology, Parkinson Research Clinic, Centre Hospitalier de Luxembourg, Luxembourg, Luxembourg; Lara Stute, Luxembourg Centre for Systems Biomedicine, University of Luxembourg, Esch-Belval, Luxembourg; Department of Neurology, Parkinson Research Clinic, Centre Hospitalier de Luxembourg, Luxembourg, Luxembourg; Pinar Alpar, Luxembourg Centre for Systems Biomedicine, University of Luxembourg, Luxembourg, Luxembourg; Piotr Gawron, Luxembourg Centre for Systems Biomedicine, University of Luxembourg, Luxembourg, Luxembourg; Soumyabrata Ghosh, Luxembourg Centre for Systems Biomedicine, University of Luxembourg, Luxembourg, Luxembourg; Jacek J. Lebioda, Luxembourg Centre for Systems Biomedicine, University of Luxembourg, Luxembourg, Luxembourg; Laurent Heirendt, Luxembourg Centre for Systems Biomedicine, University of Luxembourg, Luxembourg, Luxembourg; Enrico Glaab, Luxembourg Centre for Systems Biomedicine, University of Luxembourg, Luxembourg, Luxembourg; Armin Rauschenberger, Luxembourg Centre for Systems Biomedicine, University of Luxembourg, Luxembourg, Luxembourg; Clarissa Gomes, Luxembourg Centre for Systems Biomedicine, University of Luxembourg, Luxembourg, Luxembourg; Tainà Marques, Luxembourg Centre for Systems Biomedicine, University of Luxembourg, Luxembourg, Luxembourg; Borja G Ramos, Luxembourg Centre for Systems Biomedicine, University of Luxembourg, Luxembourg, Luxembourg; Valentin Groues, Luxembourg Centre for Systems Biomedicine, University of Luxembourg, Luxembourg, Luxembourg; Wei Gu, Luxembourg Centre for Systems Biomedicine, University of Luxembourg, Luxembourg, Luxembourg; Ahmed Hemedan, Luxembourg Centre for Systems Biomedicine, University of Luxembourg, Luxembourg, Luxembourg; Sascha Herzinger, Luxembourg Centre for Systems Biomedicine, University of Luxembourg, Luxembourg, Luxembourg; Anne Kaysen, Luxembourg Centre for Systems Biomedicine, University of Luxembourg, Luxembourg, Luxembourg; François Massart, Luxembourg Centre for Systems Biomedicine, University of Luxembourg, Luxembourg, Luxembourg; Patrick May, Luxembourg Centre for Systems Biomedicine, University of Luxembourg, Luxembourg, Luxembourg; Venkata P. Satagopam, Luxembourg Centre for Systems Biomedicine, University of Luxembourg, Luxembourg, Luxembourg; Basile Rommes, Luxembourg Centre for Systems Biomedicine, University of Luxembourg, Luxembourg, Luxembourg; Kirsten Rump, Luxembourg Centre for Systems Biomedicine, University of Luxembourg, Luxembourg, Luxembourg; Reinhard Schneider, Luxembourg Centre for Systems Biomedicine, University of Luxembourg, Luxembourg, Luxembourg; Noua Toukourou, Luxembourg Centre for Systems Biomedicine, University of Luxembourg, Luxembourg, Luxembourg; Christophe Trefois, Luxembourg Centre for Systems Biomedicine, University of Luxembourg, Luxembourg, Luxembourg; Carlos V. Moreno, Luxembourg Centre for Systems Biomedicine, University of Luxembourg, Luxembourg, Luxembourg; Maharshi Vyas, Luxembourg Centre for Systems Biomedicine, University of Luxembourg, Luxembourg, Luxembourg; Xinhui Wang, Luxembourg Centre for Systems Biomedicine, University of Luxembourg, Luxembourg, Luxembourg; Andrew Lumley, Luxembourg Institute of Health, Strassen, Luxembourg; Sopjhie Mériaux, Luxembourg Institute of Health, Strassen, Luxembourg; Lateitia Huiart, Luxembourg Institute of Health, Strassen, Luxembourg; Gloria Aguayo, Luxembourg Institute of Health, Strassen, Luxembourg; Christelle Bahlawane, Luxembourg Institute of Health, Strassen, Luxembourg; Magalie Perquin, Luxembourg Institute of Health, Strassen, Luxembourg; Manon Gantenbein, Luxembourg Institute of Health, Strassen, Luxembourg; Maura Minelli, Luxembourg Institute of Health, Strassen, Luxembourg; Tania Zamboni, Luxembourg Institute of Health, Strassen, Luxembourg; Jean-Yves Ferrand, Luxembourg Institute of Health, Strassen, Luxembourg; Sandie Boly, Luxembourg Institute of Health, Strassen, Luxembourg; Tessy Fautsch, Luxembourg Institute of Health, Strassen, Luxembourg; Jérôme Graas, Luxembourg Institute of Health, Strassen, Luxembourg; Eve Herkenne, Luxembourg Institute of Health, Strassen, Luxembourg; Alessandra Mousel, Luxembourg Institute of Health, Strassen, Luxembourg; Daniela V. Esteves, Luxembourg Institute of Health, Strassen, Luxembourg; Jean-Marc Plessaria, Luxembourg Institute of Health, Strassen, Luxembourg; Aurélie Sausy, Luxembourg Institute of Health, Strassen, Luxembourg; Sneeha Seal, Luxembourg Institute of Health, Strassen, Luxembourg; Michel Vaillant, Luxembourg Institute of Health, Strassen, Luxembourg; Charlène Verschueren, Luxembourg Institute of Health, Strassen, Luxembourg; Hermann Thien, Luxembourg Institute of Health, Strassen, Luxembourg; Integrated Biobank of Luxembourg, Dudelange, Luxembourg; Geeta Acharya, Luxembourg Institute of Health, Strassen, Luxembourg; Integrated Biobank of Luxembourg, Dudelange, Luxembourg; Wim Ammerlaan, Luxembourg Institute of Health, Strassen, Luxembourg; Integrated Biobank of Luxembourg, Dudelange, Luxembourg; Ariane Assele-Kama, Luxembourg Institute of Health, Strassen, Luxembourg; Integrated Biobank of Luxembourg, Dudelange, Luxembourg; Katy Baumont, Luxembourg Institute of Health, Strassen, Luxembourg; Integrated Biobank of Luxembourg, Dudelange, Luxembourg; Lucrèce Beckers, Luxembourg Institute of Health, Strassen, Luxembourg; Integrated Biobank of Luxembourg, Dudelange, Luxembourg; Camille Bellora, Luxembourg Institute of Health, Strassen, Luxembourg; Integrated Biobank of Luxembourg, Dudelange, Luxembourg; Fay Betsou, Luxembourg Institute of Health, Strassen, Luxembourg; Integrated Biobank of Luxembourg, Dudelange, Luxembourg; Brian De Witt, Luxembourg Institute of Health, Strassen, Luxembourg; Integrated Biobank of Luxembourg, Dudelange, Luxembourg; Ana Festas Lopes, Luxembourg Institute of Health, Strassen, Luxembourg; Integrated Biobank of Luxembourg, Dudelange, Luxembourg; Laura Georges, Luxembourg Institute of Health, Strassen, Luxembourg; Integrated Biobank of Luxembourg, Dudelange, Luxembourg; Gael Hamot, Luxembourg Institute of Health, Strassen, Luxembourg; Integrated Biobank of Luxembourg, Dudelange, Luxembourg; Estelle Henry, Luxembourg Institute of Health, Strassen, Luxembourg; Integrated Biobank of Luxembourg, Dudelange, Luxembourg; Margaux Henry, Luxembourg Institute of Health, Strassen, Luxembourg; Integrated Biobank of Luxembourg, Dudelange, Luxembourg; Alexander Hundt, Luxembourg Institute of Health, Strassen, Luxembourg; Integrated Biobank of Luxembourg, Dudelange, Luxembourg; Pauline Lambert, Luxembourg Institute of Health, Strassen, Luxembourg; Integrated Biobank of Luxembourg, Dudelange, Luxembourg; Sabine Lehmann, Luxembourg Institute of Health, Strassen, Luxembourg; Integrated Biobank of Luxembourg, Dudelange, Luxembourg; Monica Marchese, Luxembourg Institute of Health, Strassen, Luxembourg; Integrated Biobank of Luxembourg, Dudelange, Luxembourg; Maeva Munsch, Luxembourg Institute of Health, Strassen, Luxembourg; Integrated Biobank of Luxembourg, Dudelange, Luxembourg; Achilleas Pexaras, Luxembourg Institute of Health, Strassen, Luxembourg; Integrated Biobank of Luxembourg, Dudelange, Luxembourg; Lucie Remark, Luxembourg Institute of Health, Strassen, Luxembourg; Integrated Biobank of Luxembourg, Dudelange, Luxembourg; Estelle Sandt, Luxembourg Institute of Health, Strassen, Luxembourg; Integrated Biobank of Luxembourg, Dudelange, Luxembourg; Margaux Schmitt, Luxembourg Institute of Health, Strassen, Luxembourg; Integrated Biobank of Luxembourg, Dudelange, Luxembourg; Florian Simon, Luxembourg Institute of Health, Strassen, Luxembourg; Integrated Biobank of Luxembourg, Dudelange, Luxembourg; Kate Sokolowska, Luxembourg Institute of Health, Strassen, Luxembourg; Integrated Biobank of Luxembourg, Dudelange, Luxembourg; Johanna Trouet, Luxembourg Institute of Health, Strassen, Luxembourg; Integrated Biobank of Luxembourg, Dudelange, Luxembourg; Henry-Michel Cauchie, Luxembourg Institute of Science and Technology, Luxembourg, Luxembourg; Leslie Ogorzaly, Luxembourg Institute of Science and Technology, Luxembourg, Luxembourg; Christian Penny, Luxembourg Institute of Science and Technology, Luxembourg, Luxembourg; Cécile Walczak, Luxembourg Institute of Science and Technology, Luxembourg, Luxembourg; Delphine Collart, Luxembourg Institute of Science and Technology, Luxembourg, Luxembourg; Tamir Abdelrahman, Laboratoire National de Santé, Dudelange, Luxembourg; Estelle Coibion, Laboratoire National de Santé, Dudelange, Luxembourg; Marie France Pirard, Laboratoire National de Santé, Dudelange, Luxembourg; Friedrich Mühlschlegel, Laboratoire National de Santé, Dudelange, Luxembourg; Nguyen Trung, Laboratoire National de Santé, Dudelange, Luxembourg; Guillaume Fournier, Laboratoire National de Santé, Dudelange, Luxembourg; Marie Leick, Laboratoire National de Santé, Dudelange, Luxembourg; Annika Lutz, University of Luxembourg, Esch-sur-Alzette, Luxembourg; Claus Vögele, University of Luxembourg, Esch-sur-Alzette, Luxembourg; Judith Hübschen, Luxembourg Institute of Health, Strassen, Luxembourg; Christiane Hilger, Luxembourg Institute of Health, Strassen, Luxembourg; Philipp Jägi, Laboratoire Réunis, Junglinster, Luxembourg.

## Data Availability Statement

The datasets for this manuscript are not publicly available as they are linked to the CON-VINCE Study and its internal regulations. Requests to access the datasets should be directed to Rejko Krüger, mean of contact via email: con-vince@lih.lu.

## Ethics Statement

The study was approved by the National Research Ethics Committee (Comité National d'Ethique de Recherche, CNER), and by the Luxembourgish Ministry of Health under reference. The participants provided their written informed consent to participate in this study.

## Author Contributions

RK is PI of the CON-VINCE study, responsible for funding acquisition and management of the survey, investigation, writing—review, and editing. CP, VS, LP, RK, AKL, and the CON-VINCE Consortium were: involved in the conception and design of the CON-VINCE study survey and coordinated the field data collection. CP: conceptualization, investigation, and writing—original draft. LP and VS: investigation, writing—original draft and review, and editing. FR: formal analysis, writing—review, and editing. AKL: conceptualization, methodology, investigation, writing—original draft and review, editing, and supervision. All authors gave their final approval of the version to be published and agreed to be accountable for all aspects of the work in ensuring that questions related to the accuracy or integrity of any part of the work are appropriately investigated and resolved.

## Funding

The CON-VINCE Study is funded by the National Research Fund Luxembourg (FNR; 14716281/CON-VINCE/Kruger) and the André Losch Foundation (Luxembourg).

## Conflict of Interest

The authors declare that the research was conducted in the absence of any commercial or financial relationships that could be construed as a potential conflict of interest.

## Publisher's Note

All claims expressed in this article are solely those of the authors and do not necessarily represent those of their affiliated organizations, or those of the publisher, the editors and the reviewers. Any product that may be evaluated in this article, or claim that may be made by its manufacturer, is not guaranteed or endorsed by the publisher.
